# From postcard to book cover: illustrating connections between medical history and digital humanities

**DOI:** 10.5195/jmla.2019.745

**Published:** 2019-10-01

**Authors:** E. Thomas Ewing, Katherine Randall, Jeffrey S. Reznick

**Affiliations:** Professor of History, Associate Dean, and Digital Humanist, Department of History, Virginia Tech, Blacksburg, VA, etewing@vt.edu; Doctoral Candidate in Rhetoric and Writing, Department of English, Virginia Tech, Blacksburg, VA, katsbie@vt.edu; Chief, History of Medicine Division, National Library of Medicine, National Institutes of Health, Bethesda, MD, jeffrey.reznick@nih.gov

## Abstract

This article illustrates the value and impact of collaboration among scholars, archivists, and librarians working across universities and government institutions, and how changes in medium—from a born-physical photograph and printed postcard to a digital reproduction to a simultaneously born-digital and printed book—create new possibilities for scholarly analysis, interpretation, and dissemination, which in turn suggest future directions for research and engagement across fields of inquiry. In doing so, this article argues that history matters by illuminating past networks that, through humanistic inquiry, continue to connect people, ideas, and institutions in the present and into the future.

In January 1914, the Illinois Post Graduate and Training School for Nurses sent a 5⅜ by 3½ postcard (Figures 1 and 2) to Miss Eunice Hoakinson, asking her “to begin the new year right” by seeking training as a graduate nurse [1]. Over one hundred years later, the coeditors of the landmark book, *Viral Networks: Connecting Digital Humanities and Medical History* (Figure 3), selected this postcard to illustrate its cover [1, 2]. This transition from postcard to book cover represents the value and impact of collaboration among scholars, archivists, and librarians working across universities and government institutions. This process also illustrates how changes in medium—from a born-physical photograph and printed postcard to a digital reproduction to a simultaneously born-digital and printed book—create new possibilities for scholarly analysis, interpretation, and dissemination, which in turn suggest future directions for research and engagement across fields of inquiry.

**Figures 1 and 2:**
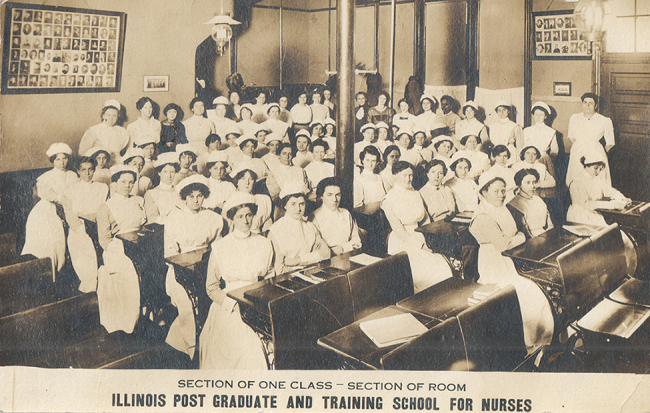

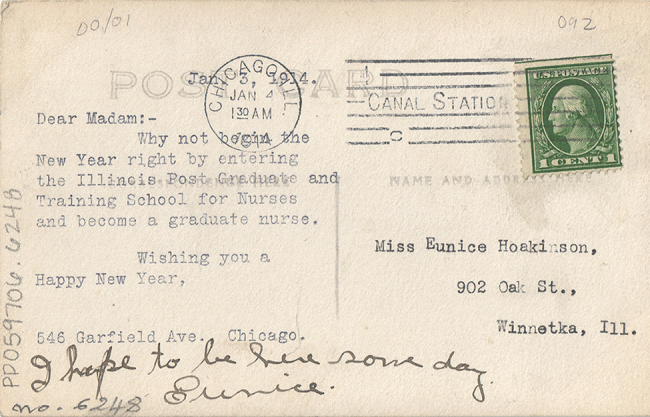
Recto and verso of the 1914 advertising postcard for the Illinois Post Graduate and Training School for Nurses Courtesy National Library of Medicine.

**Figure 3:**
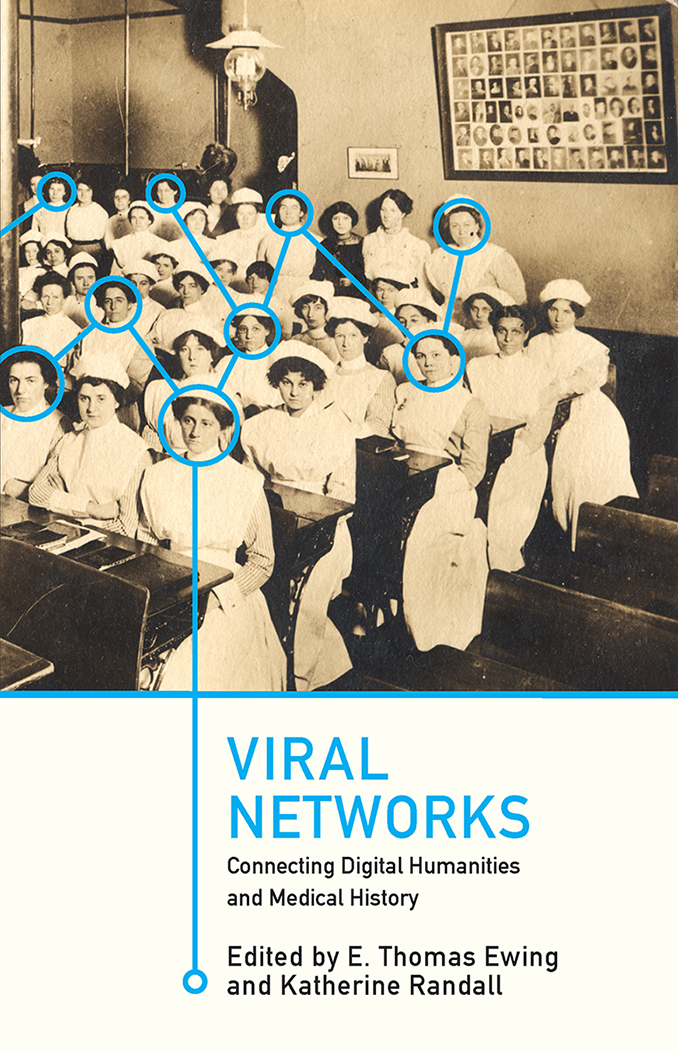
Cover of Viral Networks: Connecting Digital Humanities and Medical History Blacksburg, VA: VT Publishing, 2018.

The Illinois Training School for Nurses opened in Chicago in 1881, with 8 students who took courses in obstetrics, anatomy, physiology, and more [3, 4]. By 1914, the school enrolled nearly 170 students. During the First World War, nearly 200 graduates of the Illinois Training School for Nurses served the military in Red Cross hospitals. In 1926, the Illinois Training School for Nurses merged with the University of Chicago to meet the increased academic as well as technical requirements of nurse training, effectively closing the school. Although the closure decision came as a “distinct shock” to Jessie F. Christie, class of 1904, her reflections testified to the networked connections associated with this institution:

As I have thought it over since, I feel that it will be impossible to lose our identity as long as any of our graduates are working in the nursing field, and I get prouder day by day when I realize that our School, which was a pioneer in the nursing world, is also a pioneer in raising the standards in nursing education and making it possible for nurses to receive a scientific degree. [5]

During more than 4 decades, the Illinois Training School graduated an estimated 2,000 nurses. As Christie’s statement suggests, nurses formed professional connections with each other, with doctors, and with institutions advocating for more scientific training in the health sciences, therefore, contributing to the networks that shaped twentieth century medicine.

The photograph depicting nearly seventy nurses from the Illinois Training School for Nurses entered the collection of the History of Medicine Division of the National Library of Medicine (NLM), National Institutes of Health (NIH), in 2004, when archivists of the library acquired the Michael Zwerdling Collection of history of nursing postcards, consisting of more than 2,500 such images [6, 7]. Like many items in this vast collection, the Illinois Training School for Nurses postcard depicts nurses as professionals, placing them in a classroom setting to highlight their dedication to education and service. Yet the additional purpose of this postcard is evident in the message typed on the reverse side: “Dear Madam: Why not begin the New Year right by entering the Illinois Training School for Nurses and become a graduate nurse. Wishing you a Happy New Year.” This postcard, therefore, served as a recruiting tool, a means to bring the institution to the attention of women who might enter nursing as a profession.

One century later, scholars used—indeed, reused—a digital version of this postcard as a recruitment tool for a different purpose. In the fall of 2017, Virginia Tech partnered with NLM to recruit contributing authors for the “Viral Networks Advanced Workshop in the Digital Humanities and Medical History,” funded by the National Endowment for the Humanities (NEH) [8–10]. Because this photograph clearly showed people working in a medical context, it encouraged potential contributors to think about viral networks in terms of human connections to disease, medicine, and health.

On October 22, 2017, the image was tweeted, with the text: “Committed to using #digitalhumanities to advance scholarship? Proposals due 11/18 viral networks workshop.” This particular tweet soon earned 3,935 impressions and 42 engagements, illustrating how the transition from a born-physical artifact to a twenty-first-century digital copy circulating in a social network engaged new audiences while evoking the same themes—education and recruitment—that the original artifact did a century earlier. Digital networking reanimated this historical image not merely as a digitized object, but as one purposefully selected, curated, and circulated to achieve a new objective and advance the humanistic enterprise defined by a singular yet multifaceted initiative.

As the coeditors of the *Viral Networks* book worked with VT Publishing to select an illustration for the cover of the publication, they sought an image to convey the shared themes of the chapters while also respecting the diversity of chronologies, geographies, and methods covered by their authors. In addition to illustrating the volume’s focus on medical history, the cover also needed to emphasize how individuals across places and times constructed and contested knowledge about disease and health. Finally, the cover needed to demonstrate that people constitute networks, not only as nodes that connect edges, to use the terminology of network analysis, but fundamentally as the actors whose practices, intentions, attitudes, and interactions give meaning, value, and context to networked connections.

This photograph in the 1914 postcard was familiar to the editors from the tweet sent in October 2017, and they selected it as the best possible image to meet the stated objectives. In the design process, they cropped the image and purposely reversed it to enhance its aesthetic quality by aligning the pole in the middle of the photograph with the spine of the book. The photograph itself indicated a valuable connection to medical history, both in the aged appearance, which was preserved in its digital format, and in the composition of the photo itself, which featured old-fashioned nursing uniforms and unsmiling subjects often associated with early photography. To highlight the potential for a networked connection among these individuals, circles and lines in cyan blue were added to connect the faces of nurses to each other.

Using “new” digital tools to overlay an imaginary network on an “old” medium changes the story and purpose of the photograph. Originally a recruitment tool—both in its analog twentieth century use and a twenty-first century tweet—the cover, therefore, demonstrates how new technologies can make historical networks visible while maintaining, if not augmenting, a humanistic focus on the people involved.

In December 2018, VT Publishing released *Viral Networks* both in a print version, available in black and white or full color, and in an electronic open-access version [11, 12]. Publishing the volume in the latter format was—and remains—consistent with the missions of the partner organizations to make knowledge at the intersection of medical history and digital humanities widely and easily available to all. The photograph of the Illinois Training School for Nurses, therefore, circulates as the book cover in both digital and print versions, including copies sent to medical history libraries to add to their circulating collections.

In April 2019, the NLM History of Medicine Division convened, broadcasted globally, and subsequently archived the livestream of “Viral Networks, Reconnected: A Digital Humanities/History of Medicine Research Forum” to continue the dialogue about innovative research methods using knowledge and tools of the digital humanities [13, 14]. Featured prominently throughout these proceedings, the postcard of the Illinois Training School for Nurses continued to achieve an extended presence and scholarly usefulness via the Internet, on social media, in libraries, and in the hands of readers of the associated book.

Yet, this postcard contains an additional detail, one that was initially elusive but, through further research, became compelling. The reverse of the postcard included a handwritten note: “I hope to be here some day. Eunice.” The Winnetka directory for 1914 revealed that Eunice Hawkinson lived at 902 Oak Street (though her name on the postcard was misspelled as Eunice Hoakinson) [15]. The directory also listed her occupation as “nurse,” suggesting her handwritten note was an expression of a desire to increase her qualifications in this chosen profession. Despite her stated desire to attend the Illinois Training School for Nurses, the list of alumni for subsequent classes did not reveal a student by either name (Hoakinson or Hawkinson). The 1917 Winnetka directory still listed Eunice Hawkinson at 902 Oak Street, yet her occupation was “maid” [16]. Two years later, on May 3, 1919, Eunice Hawkinson married Conrad Carlson [17], and the 1920 Census listed them living in New Trier [18]. Twenty years later, a Eunice Carlson, now widowed, was recorded in the 1940 Census as living in Chicago [19]. After 1940, no further records have been discovered that reveal subsequent events in her life.

Eunice Hawkinson and the Illinois Post Graduate and Training School for Nurses, therefore, appear to have two connections: first, a recruitment postcard, which prompted a very personal expression of interest from Eunice Hawkinson, yet there was no evidence of any further intersections during the course of her lifetime; and second, this article, which establishes connections among an individual, an institution, an artifact, and an edited volume. In deliberately choosing to circulate this image, the editors by inevitable association also chose Eunice Hawkinson’s note and her story, understanding the situatedness of both networks and artifacts.

Recognizing the contingency as well as complexity of connections is consistent with the themes of the book. Networks connect people, ideas, and processes, yet it is also important to recognize gaps, omissions, and absences within the networks that must also be subject to analysis. From this critical perspective, the search to find more information about the Eunice “Hoakinson” listed on the postcard reminds us that any kind of humanities analysis is an effort to understand, uncover, and contextualize what knowledge is present, what is missing, and, most importantly, what may be connected. History matters by illuminating past networks that, through humanistic inquiry, continue to connect people, ideas, and institutions in the present and into the future.

**E. Thomas Ewing, PhD**, etewing@vt.edu, Professor of History, Associate Dean, and Digital Humanist, Department of History, Virginia Tech, Blacksburg, VA

**Katherine Randall**, katsbie@vt.edu, Doctoral Candidate in Rhetoric and Writing, Department of English, Virginia Tech, Blacksburg, VA

**Jeffrey S. Reznick, PhD**, jeffrey.reznick@nih.gov, Chief, History of Medicine Division, National Library of Medicine, National Institutes of Health, Bethesda, MD
